# A review of 10 years of human microbiome research activities at the US National Institutes of Health, Fiscal Years 2007-2016

**DOI:** 10.1186/s40168-019-0620-y

**Published:** 2019-02-26

**Authors:** NIH Human Microbiome Portfolio Analysis Team, Lita Proctor, Jonathan LoTempio, Aron Marquitz, Phil Daschner, Dan Xi, Roberto Flores, Liliana Brown, Ryan Ranallo, Padma Maruvada, Karen Regan, R. Dwayne Lunsford, Michael Reddy, Lis Caler

**Affiliations:** National Institutes of Health, Bethesda, MD USA

**Keywords:** Microbiome, Microbiota, Metagenomics, Human disease, Animal models, NIH funding, Human Microbiome Project (HMP), Fast Track Action Committee on Mapping the Microbiome (FTAC-MM), National Microbiome Initiative (NMI)

## Abstract

The National Institutes of Health (NIH) is the primary federal government agency for biomedical research in the USA. NIH provides extensive support for human microbiome research with 21 of 27 NIH Institutes and Centers (ICs) currently funding this area through their extramural research programs. This analysis of the NIH extramural portfolio in human microbiome research briefly reviews the early history of this field at NIH, summarizes the program objectives and the resources developed in the recently completed 10-year (fiscal years 2007–2016) $215 M Human Microbiome Project (HMP) program, evaluates the scope and range of the $728 M NIH investment in extramural human microbiome research activities outside of the HMP over fiscal years 2012–2016, and highlights some specific areas of research which emerged from this investment. This analysis closes with a few comments on the technical needs and knowledge gaps which remain for this field to be able to advance over the next decade and for the outcomes of this research to be able to progress to microbiome-based interventions for treating disease and supporting health.

**Electronic supplementary material:**

The online version of this article (10.1186/s40168-019-0620-y) contains supplementary material, which is available to authorized users.

## Introduction

### Brief introduction to early period of human microbiome research field

A number of pioneering studies, particularly in the early 2000s, helped lay the foundation for this field and inform the development of the HMP program. Though by no means exhaustive, examples include the extensive work on the diversity of oral pathogenic and nonpathogenic bacteria using 16S rRNA gene sequence analysis [1, 2]. This work later led to the development of a major resource for the field, the Human Oral Microbiome Database (HOMD) [3].

New approaches to the study of the gut microbiome led to seminal contributions in our understanding of the role of the gut microbiome in human biology, e.g., [4] and in disease, e.g., [5]. The germ-free humanized mouse model was developed during this early period and became a broadly adopted model for the study of the gut microbiome [6]. Early work also led to foundational knowledge on the role of birth mode, e.g., [7] and breast milk, e.g., [8] in the development of the infant gut microbiome. Another important development was the recognition that some host disease phenotypes were associated with the gut microbiome, a property which could be transferred when the gut microbiome was transferred from the diseased donor to a recipient host, e.g., obesity [9].

DNA sequencing technologies including 16S rRNA gene-based amplicon sequence analysis and whole genome shotgun metagenomic analysis were first used by ecologists in the 1980s studying the microbial communities of oceanic and forest ecosystems. A National Research Council report [10] described the power of these molecular approaches to reveal astonishing microbial diversity and genetic potential in many different habitats and hosts. Researchers in the biomedical field then adopted this approach to explore the microbial diversity of the human body. These early metagenomic studies examined the GI tract and found tremendous complexity and metabolic potential encoded in the human gut microbiota [11, 12].

### Overview of HMP

By the mid-2000s, DNA sequencing costs had decreased sufficiently for NIH to mount an initiative to study the microbial communities associated with the human body. In fiscal year 2007 (FY2007),^1^ the NIH Office of Strategic Coordination launched the 10-year $215 M HMP program [13]. From its inception, the HMP was designed as a community resource program to develop broadly available, and rapidly released computational and statistical tools, analytical and clinical protocols, and reference datasets in order to serve as a catalyst for this emerging field.

The HMP program had two phases. The first phase (FY2007–2012) was designed to conduct a survey of microbial communities from five major habitats of the human body (oral, skin, nares, GI tract, and urogenital tract) and to evaluate whether a characteristic microbial community was associated with a specific host health status [14]. This phase of HMP developed the clinical protocols for sampling the microbiome in these five body regions [15]. In addition, HMP created reference catalogs of microbial (e.g., bacterial, archael, bacteriophage, viral, and fungal) genome sequences, which were deposited at NCBI [16] with live cultures for many of these reference genomes deposited at ATCC [17]. Computational tools and reference 16S rRNA gene and whole genome shotgun metagenomic sequence datasets were also developed for use by the broader research community; these datasets and tools are available through the HMP Data Analysis and Coordination Center (DACC) [18].

One key HMP phase 1 study analyzed the microbial communities across these 5 habitats for a cohort of 300 healthy American adults and found that common metabolic pathways were associated with taxonomically dissimilar microbial communities, which indicated that microbial community makeup alone may not serve as a biomarker for host phenotype [19]. Other HMP human cohort studies which investigated the microbiomes of subjects with specific GI, oral, or urogenital diseases noted that specific microorganisms and/or specific microbial metabolic pathways, and not total community composition, differed from the healthy controls. For example, this trend was observed in HMP studies of the gut microbiome in Crohn’s disease patients [20, 21].

Outcomes from the first phase led to the recognition that deeper analysis of the microbiome, beyond community composition, was needed to understand the functional contributions of these microbial communities to human health and disease and to evaluate whether specific microbial functional properties were associated with specific host phenotypes. Further, there was a clear need to evaluate the biological properties of host and microbiome together in order to study cause and effect relationships between host and microbiome. The second phase (FY2013–2016) of this program, known as the integrative HMP (iHMP), was designed to create an integrated dataset of the biological properties of both the microbiome and host over time, in a series of disease cohorts, as a resource for the broader research community.

Three iHMP clinical studies were developed as models of microbiome-associated conditions, including one on preterm birth with a focus on the vaginal microbiome of the mother [22], one on inflammatory bowel disease with a focus on the GI microbiome [23], and one on type 2 diabetes with a focus on both the GI and nasal microbiomes [24]. These projects analyzed microbial community compositions as well as the transcriptomes and proteomes of the microbiomes, along with the global metabolome and other key properties such as immune and clinical markers from the subjects in longitudinal cohort studies [25]. These studies will be published as a part of an iHMP collection of papers [in press, Nature]. The HMP DACC also houses the integrated datasets and computational tools from these three studies [18].

### Motivation for portfolio analysis of NIH extramural human microbiome research

In 2015, the Office of Science and Technology Policy (OSTP) under the Obama Administration White House chartered a committee of the federal government agencies that conduct or support research to complete a survey of all federally-supported microbiome research over FY2012–2014; this was known as the Fast-Track Action Committee on Mapping the Microbiome (FTAC-MM). The FTAC-MM analysis included a broad collection of studies of the microbial communities of plants, animals, humans, as well as marine and terrestrial ecosystems that used microbial community-level ‘omics approaches and comprised research support from 16 government agencies. The FTAC-MM identified an investment of $920 M in both intramural and extramural microbiome research across these 16 agencies over FY2012–2014 [26].

To gain a deeper understanding of federal support for human health-focused microbiome research and to identify key research gaps and opportunities, the Trans-NIH Microbiome Working Group (TMWG) [27] held a three-day NIH-wide microbiome workshop in 2017; a  report from this meeting is published in this issue [28]. Further, a committee, the NIH Human Microbiome Portfolio Analysis Group (NHMPAG), formed from the TMWG [27], initiated an in-depth analysis of research supported through the NIH extramural programs. To this end, the NHMPAG developed a customized query of the NIH grant database to identify candidate grants over five fiscal years (FY2012–2016). This period was selected because it overlapped with the FTAC-MM analysis period and because it included the last 5 years of the HMP program. The results from the initial query were manually curated to confirm that the specific aims of the applications specified microbiome research and to apportion the funding so that only the specific support for microbiome research was included in the subsequent analysis. This approach differed from the FTAC-MM analysis, which included the entire grant budget for each award, so the NHMPAG approach serves as a more specific estimate of NIH extramural human microbiome research.

The NHMPAG adopted the FTAC-MM definition of a microbiome as “a multi-species population or community of microbes in a specific host or environment” where the term “microbes” included bacteria, fungi, archaea, eukaryotic viruses, and bacteriophage [26]. Further, the FTAC-MM definition of microbiome research as “the study of these communities with regard to their composition, structure and function as well as studies of microbe-microbe interactions or interactions with their hosts in human health or disease” was also adopted [26]. These definitions helped to minimize inclusion of research on specific pathogens or on specific aspects of the immune system (both of which encompass a very large fraction of the NIH research budget) and so minimize the unintended inflation of support for microbiome research in the NHMPAG analysis. The NHMPAG analyzed the manually curated list of grants by type of study, microbiome properties collected, and other features of the research. Since grant award periods can vary greatly, the results of this analysis are reported in 1-year increments, which are called “projects.” The data were anonymized with regard to specific investigators, institutions, ICs, or funding opportunity announcements (FOAs). Particular emphasis was placed on the types of microbial and microbiome properties studied and the approaches and methods employed to conduct the study. In addition, the analysis evaluated whether the research resources developed in the Human Microbiome Project were leveraged by these other studies. Finally, a summary of HMP and nonHMP research support over the 10 years of the HMP program (FY2007–2016), derived from earlier NIH portfolio analyses, was included to provide a comparison with the NHMPAG analysis of microbiome research activities over the more recent 5 years (FY2012–2016).

A more detailed description of the methods used to conduct this portfolio analysis as well as the Institute and contact information for the writing team members can be found in Additional file 1. Here, we summarize the key findings that emerged from this portfolio analysis.

## Results

### HMP catalyzed microbiome research at NIH

**Fig. 1:**
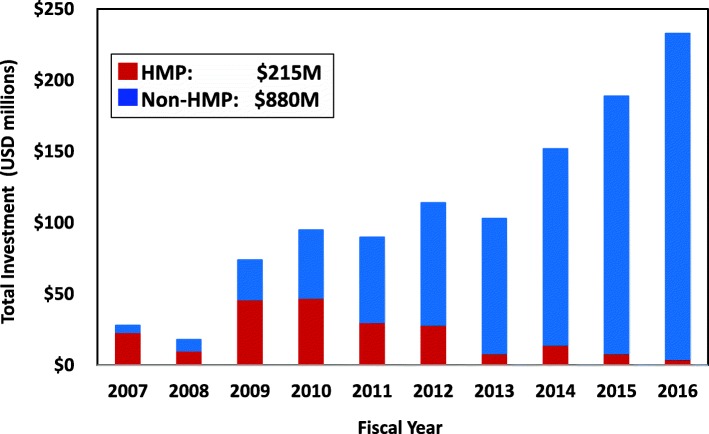
Annual NIH investment in human microbiome research, FY2007–2016. NIH investment for extramural human microbiome research support depicted as annual sums of all microbiome projects included in NHMPAG portfolio analysis; data for FY2007–2012 period taken from earlier portfolio analyses. The annual sum of HMP program projects shown separately from the annual sum of all non-HMP supported projects. Over FY2007–2016, the HMP awards totaled $215M and all non-HMP awards totaled $880M

Prior to the start of the HMP program, support for the human microbiome field at NIH was modest. Five NIH ICs were supporting human microbiome research activities at an estimated level of $5M/year (data not shown). In FY2007, NIH began a major initiative in this area with the establishment of the 10-year HMP program and from FY2007 to 2010, HMP represented over half of total NIH support for this field (Fig. 1). However, support for microbiome studies in the individual ICs grew quickly, outpacing HMP support by FY2011. By FY2012, NIH support for human microbiome research outside of the HMP eclipsed the annual HMP investment and reached or exceeded $100M/year.

Over 10 years (FY2007–2016), NIH provided approximately $880M of support in this research area, above the $215M invested in the HMP program (Fig. 1). These nonHMP-funded research activities were primarily investigator-initiated projects since only a few targeted FOAs for human microbiome research were released during this period. The HMP supported 65 investigators (defined as the principal investigator or as the main contact in a multi-PI grant) over the life of the program. As the number of NIH ICs and amount of support increased, the number of total investigators who were not a part of the HMP pursuing studies in this area significantly increased, particularly during FY2012–2016. In FY2012, there were 157 principal investigators outside of the HMP receiving microbiome-related awards from 15 NIH ICs. This total included individual investigator, center, and training grants. This researcher pool more than doubled by FY2016 to 391 investigators across 21 ICs. The researcher pool totaled 1049 investigators over this 5-year period with an average of 63% of this researcher pool made up of individual investigators. Furthermore, much of this increase in microbiome research support occurred during a period (ca. FY2010–2015) when the total NIH appropriations had either not increased or, in some years, had even declined [29], further demonstrating the rapid growth of interest in this field.

**Fig. 2:**
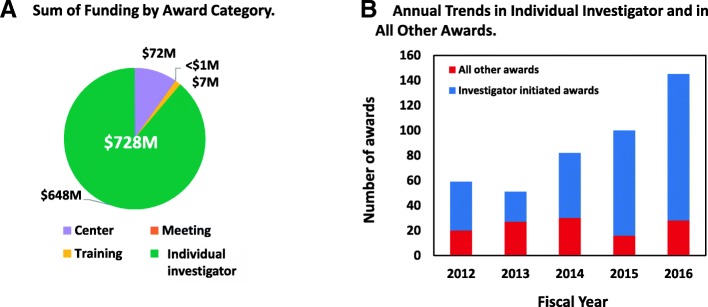
NIH human microbiome grant awards by award type, FY2012–2016. **a** Depicts the sum of all NIH microbiome grants over fiscal years 2012–2016, which totaled $728M. These awards were subdivided into the four award categories of individual investigator-initiated, center, training, and meeting grants. **b** Depicts annual trends in the number of individual investigator-initiated awards versus awards for all of the other award categories combined

A more detailed analysis of NIH-supported microbiome research activities over FY2012–2016 revealed a total of $791M of support for this area; $63M of this total was for the HMP program (Fig. 2). Of the $728M nonHMP funds identified over this period, the vast majority (88%) of the funds supported individual investigator-initiated grants (Fig. 2a); the remainder supported microbiome research activities in center grants (10%), and a small amount of training (1%) and meeting activities (1%) related to the microbiome. As another point of comparison, the majority of these awards were in response to the omnibus NIH program announcements, which invite unsolicited investigator-initiated applications. This trend remained throughout the 5-year period (Fig. 2b). Further, investigator-initiated microbiome projects expanded in number, particularly from FY2014 onward. This rapid expansion suggested that the HMP-supported clinical, analytical, and computational resources and databases were leveraged by the larger research community.

Further, of the approximately 2700 total projects which comprised the NHMPAG analysis, over half (average 68%) of the total award budget of the grants were applied to the microbiome component of the study (data not shown). This relatively high percentage of award dollars applied to microbiome research suggested that the microbiome component of a grant was not simply an “add-on” feature of the research but instead constituted a central feature of the proposed research.

Finally, we noted that several NIH Offices contributed co-funding support toward these projects. Approx. 800 of the 2700 projects received co-funding from the Office of AIDS Research (OAR), the Office of Research on Women’s Health (ORWH), and the Office of Dietary Supplements (ODS) for a total of $132M in co-funding provided over these five fiscal years, with most of this OAR support (data not shown).

### NIH-funded microbiome research focuses on both health and disease

**Fig. 3:**
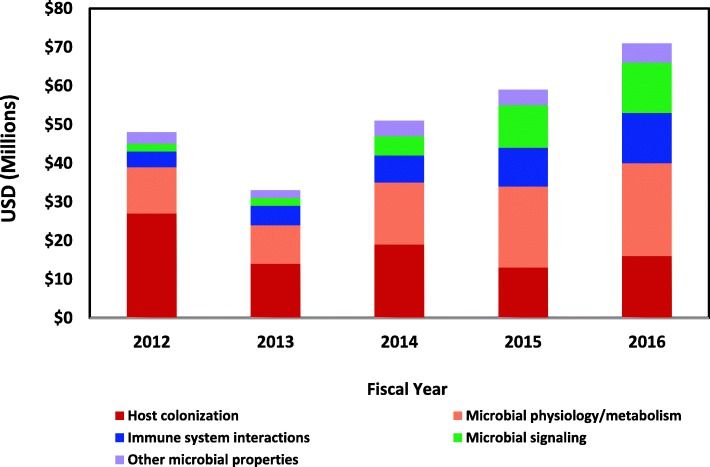
Annual trends in projects on the basic biology of and host/microbe interactions within the human microbiome, FY2012–2016. Annual trends in non-disease focused microbiome projects depicted; the sum of these projects over fiscal years 2012–2016 was $262M. These projects have been subdivided into four broad topics which included studies of microbial colonization of the host, physiology, and metabolism of microbial members of the microbiome, host immune system interactions with microbes, and microbial signaling between members of the microbiome and between host and microbe. Projects that focused on three additional broad topics of microbial properties were combined and depicted under “Other properties”

**Fig. 4:**
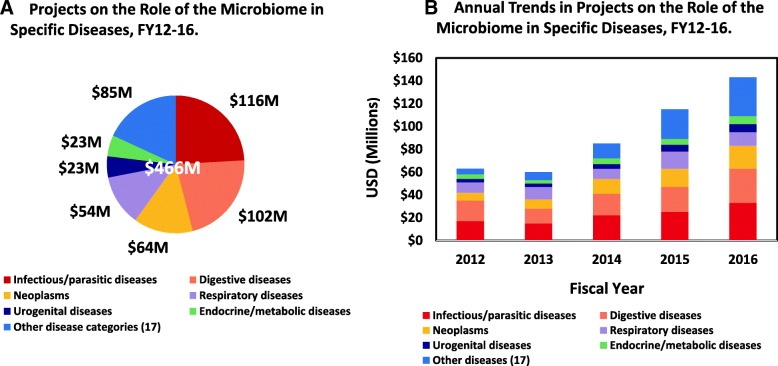
NIH projects on the role of the microbiome in specific diseases, FY2012–2016. **a** Depicts the sum of all disease-focused microbiome projects over fiscal years 2012–2016, which totaled $466M. These projects have been subdivided into six major ICD-10 chapter-level disease categories which included A00-B99 infectious/parasitic diseases, K00-K95 digestive diseases, C00-D49 neoplasms, J00-J99 respiratory diseases, N00-N99 genitourinary diseases, and E00-E89 endocrine/metabolic diseases. Projects that focused on 17 additional ICD-10 chapter disease categories were summed as “Other.” **b** Depicts annual trends in these disease-focused microbiome projects

Though many of the individual studies in this portfolio analysis addressed the association of the microbiome in a variety of diseases, not all of this research focused on disease. In fact, over this 5-year period, a significant amount of this research effort was on the study of the biological properties of the microbiome or on fundamental host/microbiota interactions in health. Approx. 36% ($262M) of the total funding over FY2012–2016 ($728M) were studies of the basic biology of the microbiome (Fig. 3) while 64% ($466M) of the support was for studies on the relationship of the microbiome in various diseases (Fig. 4).

In this analysis, those projects that focused on the basic properties of the microbiome or on host/microbiome interactions were binned by the NHMPAG into one of seven major areas of biological emphasis. These seven areas included factors related to the microbial colonization of the host, studies of microbial community-level physiology and metabolism, interactions of the host immune system with the commensal microbiota, studies of microbial signaling within the microbiome communities, as well as a broad array of studies investigating other aspects of the microbiome or microbiota or host/microbiota interactions.

Of the $262M invested in non-disease studies of the microbiome, the largest fraction (approx. 33%) of the work focused on factors related to microbial colonization of the host (Fig. 3). However, interest in this topic appeared to decrease over this 5-year period with approx. 55% of the funding in FY2012 devoted to this topic decreasing to 22% of the funding by FY2016. At the same time, interest in other “basic” questions about microbial community biology expanded. For example, support for the study of microbial signaling within the microbiome and with the host and related topics increased over fourfold from less than 4% of the funding in FY2012 to 18% of the funding in FY2016. This increased interest in microbial signaling and in other biological features of the microbiome was due partly to the increase in the availability of high throughput ‘omic analysis methods and in new experimental approaches. This trend also reflected how quickly the field expanded focus from characterization of the microbial communities to functional studies of these communities.

As a basis for classifying the diseases studied in these microbiome projects, the NHMPAG used the W.H.O. International Classification of Diseases, version 10 (ICD-10) [30]. Over FY2012–2016, diseases in six of the ICD-10 chapter-level disease categories comprised the bulk of the research on the relationships between the microbiome and various diseases (Fig. 4a). These six categories, which included ICD-10 chapters A00-B99 infectious and parasitic diseases, K00-K95 digestive diseases, C00-D49 neoplasms, J00-J99 respiratory diseases, N00-N99 genitourinary diseases, and E00-E89 endocrine, nutritional, and metabolic diseases, summed to approx. 82% of total support in disease-related microbiome projects over FY2012–2016. The remaining 18% included support for microbiome studies in an additional 17 other ICD-10 disease categories.

The relative proportion of support for the study of the microbiome in the six main ICD-10 disease categories noted above remained steady throughout these five fiscal years (Fig. 4b). However, the study of the relationship of the microbiome in the other 17 ICD-10 disease categories showed the greatest change, as evidenced by an over sevenfold increase in support for microbiome studies in these other disease categories from FY2012 to FY2016. This trend demonstrated the rapidly expanding interest in the role of the microbiome, either as an indicator, precursor, causal factor, or exacerbator of disease, across an increasingly greater variety of disease classes.

### NIH microbiome research relied on both human cohorts and animal models

**Fig. 5:**
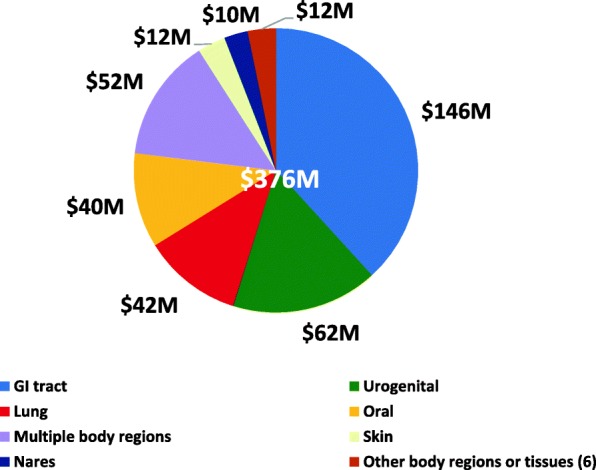
Body regions investigated in microbiome projects with human cohorts, FY2012–2016. This figure depicts human cohort studies of the microbiome, which totaled $376M over fiscal years 2012–2016. Six major body regions were investigated in these studies with human cohorts, and included gastrointestinal tract, urogenital tract, lung, oral, nares, and skin. Some cohort studies included the simultaneous study of multiple body regions and were noted as “Multiple body regions.” Those studies which focused on six additional body regions or tissues (blood, ear, eye, liver, cardiovascular system, central nervous system) were combined into ‘Other’

In general, most NIH research is conducted with human cohorts or animal models or a combination thereof. Over FY2012–2016, more than half ($376M) of the total funding ($728M) invested in projects that used human cohorts to investigate the microbiome (Fig. 5). Approx. three-fourths ($290M) of this work focused on the microbiomes of one of four major body regions, GI tract, urogenital tract (primarily vaginal), oral, and lung with studies of the GI tract microbiome representing about 40% of this total. An additional 14% was invested in simultaneous studies of multiple body regions in human subjects, often as a combination of the GI tract with one or two other body sites. Almost all of these GI tract studies relied on stool as the gut microbiome sample. Approximately 3% was invested in the study of the skin microbiome and another 3% in the nares microbiome of human subjects. The remaining funding (3%) supported microbiome studies of six other body regions or tissues, including blood, cardiovascular system, central nervous system, ear, eye, and liver.

Approximately $231M over FY2012–2016 was invested in animal model studies of the microbiome; almost 90% of these involved vertebrate models, such as mouse, rat or rabbit, and a few non-human primate models (data not shown). Unlike the human cohort studies, most of these studies (83%) focused on the GI tract microbiome. Most of the additional funding (17%) in animal model studies investigated the microbiomes of other body regions or tissues (blood, ear, eye, liver, skin, urogenital, lung, oral, and nares) or of multiple body regions, generally GI tract in combination with other body regions.

Finally, about 11% of total support (or $81M) for these projects used a combination of human cohort and animal models (data not shown). In many cases, results from the human cohort studies were verified in the animal models or the animal models were used to carry out follow-on mechanistic research. The smallest fraction (5%) used available human cohort data, such as from the HMP healthy cohort study, or animal model data for in silico modeling or other computational approaches to investigate the microbiome.

### Which properties of the microbes and microbial communities were studied?

This portfolio analysis includes an assessment of the various properties of the microbiota and microbial communities studied in these projects. This assessment was conducted at two levels of detail. The first pass was to evaluate the general features of host-microbiome community interactions studied in these projects. These features were classified by the NHMPAG into one of five broad topics: larger community interactions, specific microbe-microbe interactions within microbial communities, the role of a specific microbe in the microbial community, the role of microbial products produced in microbial communities; biofilms were a fifth category. On a more detailed level, the analysis evaluated the primary microbial member or microbial property under study in each project. The seven microbial categories at this level of the evaluation included bacteria, archaea, viruses/bacteriophage, fungi, specific microbial products, specific microbes (such as a specific bacterial species), and finally, those studies which investigated the interactions of specific multiple microbial members in the microbiome.

Of the general properties, larger community interactions were studies which had a primary focus on the whole community, typically done through 16S rRNA gene sequence-based analysis of microbial community composition. Microbe-microbe interactions included studies that examined interactions between different microbial groups, such as between different specific commensal species in a microbial community or the interaction of a known pathogen with specific commensal species. In projects that included a pathogen, only those studies that investigated pathogen interactions with the larger microbial community or in interactions with a specific commensal microbe were included in this analysis. Studies that focused on specific microbes were included only if the microbe was used as a model for a commensal member within the larger community. Since many microbial products are important signaling molecules and host energy substrates (e.g., short-chain fatty acids), projects with a primary focus on microbial metabolism and microbial products were separately categorized. Finally, even if a project addressed more than one of these general topics, only one primary topic was attributed to each project.

**Fig. 6:**
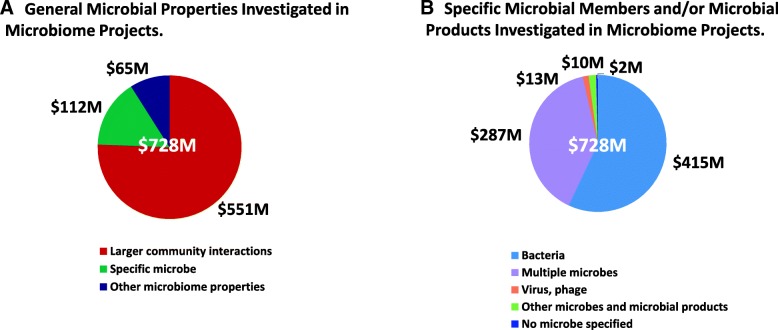
Microbial features investigated in the microbiome projects, FY2012–2016. **a** Depicts the general microbial properties investigated in the projects, which were subdivided into three broad categories of larger microbial community interactions, specific microbe-microbe interactions in the microbial community and other microbiome properties (i.e., microbe-microbe interactions, biofilms, microbial products). **b** Depicts the specific microbial member(s) of the microbiome and/or microbial products which were the primary focus of the projects. The four specific categories included bacteria, bacteriophage or eukaryotic virus, interactions between multiple members in the microbiome, and other microbes (i.e., archaea or fungi) and/or specific microbial products. Some projects did not specify a particular microbe in the study

Over 75% of total funding ($728M) during this 5-year period supported studies which focused on larger community interactions in the microbiome, regardless of whether the studies focused on disease (Fig. 6a). Approx. 15% of these activities focused on the study of specific microbes in the microbiome, either because a commensal microorganism appeared to be associated with a specific disease or, in some cases, because specific microbes served as a model for commensal microorganisms in the microbiome. The remaining 9% focused on the study of biofilms or on microbial products.

On a more detailed level, this analysis also assessed the primary microorganism or microbial product under study. Studies which investigated multiple microbes in the microbiome, such as the interactions of several specific bacterial species in a microbiome or the interactions of bacteria and fungi or bacteria and viruses in a microbiome, were also evaluated. Finally, even if a project studied more than one microbial member or microbial property, this analysis included only one primary microbial feature for each project.

Regardless of whether there was a disease focus, over half of the total funding over this 5-year period ($728M) supported studies which focused exclusively on the bacterial members of the microbiome, either as studies of bacterial community composition in relation to host health status, as studies of specific bacterial species within the larger bacterial community, or on studies of a specific commensal bacterium (Fig. 6b). An additional 40% of the support for these studies focused on multiple members of the microbiome, either because they investigated interactions of different specific bacterial members or, in some cases, on the interactions of bacteria and fungi or of bacteria and viruses. A small fraction (1%) of these studies had a primary focus on the viral or bacteriophage component of the microbiome. A similarly small fraction (2%) focused primarily on other microbial features such as the fungi, archaea or microbial products.

Though there have been a number of reference culture collections of nonclinical isolates produced by the HMP and by other researchers (e.g., HOMD), a surprisingly small number of known commensal microorganisms were studied over this period (Table 1). Most of these commensals are isolates from the intestinal microbiota and included a number of genera and/or species found associated with the gut lumen or in the lymphoid tissue of the gut lining in the large colon. A small number of skin, oral, and vaginal commensals were also the subjects of study. Further, several of these projects examined the interactions of these commensals with pathogens, such as *Salmonella enterica*, *Enterococcus faecalis*, *Porphyromonas gingivalis*, *Clostridium difficile*, or *Staphylococcus aureus*.

**Table 1:** Microorganisms, microbial substrates, and microbial products under study, FY2012–2016

Body region	Commensal microbial isolates	Microbial products		Microbial substrates
		Cellular	Metabolic	
GI tract—lumen	*Bacteroids fragilis*	Lipopolysaccharide	Indole	Fiber
	*B*. *thetaiotamicron*	Flagellin	Short-chain fatty acids (butyrate, acetate, proprionate)	Human milk oligosaccharides
	*B*. *ovatus*	Bacteriocins	Corrinoids (B vitamins)	Plant pectin glycans
	*Bacteriodes* spp.	Amyloids	Hydrogen sulfide	Fructans
	*Methanobrevibacter smithii*	Polysaccharide A	Secondary bile acids	Host mucin glycans
	*Escherichia coli*	Sphingolipids	Pyrazinones, dihydropyrazinones	Polyphenols
	*Oxalobacter formigenes*	CRISPRs	Trimethylamine N-oxide	Inulin
	*Akkermansia mucinophila*	Type IV pili	Thiopeptides	
	*Enterococcus faecium*		Oxalate	
	*Candida albicans*			
GI tract—lymphoid	*Alcaligenes* spp.			
	*Achromobacter* spp.			
	*Ochrobacter* spp.			
	*Bordetella* spp.			
	*Bifidobacterium adolescentis*			
Skin	*Corynebacterium* spp.			
	*Staphylococcus epidermidis*			
Oral	*Proprionibacterium* spp.			
Vagina	*Clostridiales* spp.			
Nares	*S*. *aureus*			
Body-wide	*Neisseria* spp.			

This table summarizes the commensal microorganisms, microbial growth substrates, and bioactive microbial products which were the subject of study in the microbiome-related projects over FY12–16. The first two columns indicate the body region from which the commensal microorganism was isolated, and the name of the isolate. The next two columns list the microbial products which were the subject of study and which are either cellular in nature or a byproduct of microbial metabolism. The final column lists the microbial growth substrates which were a subject of study

There was also interest in the metabolism and role of specific bioactive microbial products, whether as cellular components or metabolic products (Table 1). The relatively small number of cellular products under study during this period included cell membrane components (e.g., lipopolysaccharides, polysaccharide A, sphingolipids, type IV pili), antibacterial proteins (e.g., bacteriocins), flagellar proteins, or biofilm components (e.g., amyloids). Many of these cellular products were studied because of their role in eliciting host immune system responses. There were also a number of specific bioactive microbial metabolic products studied (Table 1). These metabolic products were studied because they serve as signaling molecules between microbial members or between host and microbe, for example, short-chain fatty acids. Some are antimicrobial molecules, such as thiopeptides, pyrazinones, or dihydropyrazinones. CRISPRs were studied because of their role in bacteriophage defense by bacteria. Finally, a small number of microbial growth substrates were the subject of study (Table 1). These primarily included specific constituents of the human diet or of human breast milk. In a few of these projects, microbial metabolism of host mucins was also studied.

### What kinds of microbiome and related data were collected in these projects?

High throughput sequencing and other modern ‘omics methodologies, such as transcriptomics, proteomics, and metabolomics, have enabled the study of microorganisms at the community level. In addition, computational approaches have changed the way microbial communities are studied. In this portfolio analysis, the primary methods used to investigate the microbiome were evaluated and classified as data derived from sequence-based analyses, multi-omic analyses, immunological analyses, computational analyses, or combinations thereof. In addition, trends in these analyses were evaluated to determine if laboratory methodologies were changing the types of results being reported over this period.

**Fig. 7:**
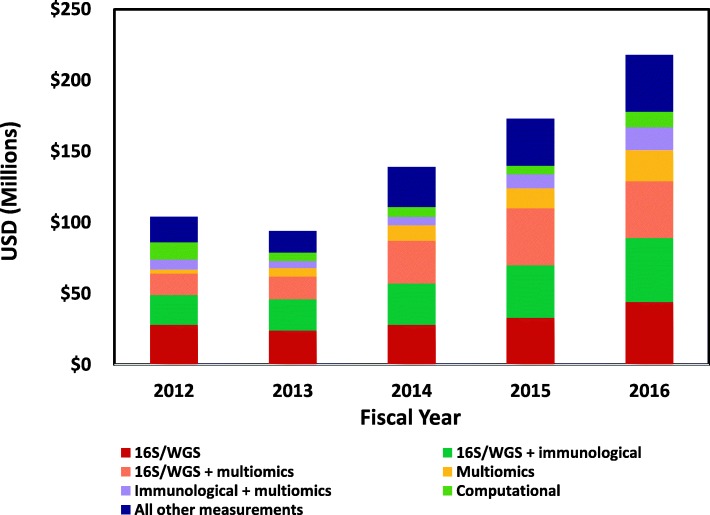
Annual trends in the types of microbiome and related data collected in projects, FY2012–2016. This figure depicts trends in the primary microbiome and related data collected in these projects. These data have been categorized into one of six main types and include data from 16S rRNA gene sequence analysis, data from 16S analysis combined with data from immunological analyses, data from 16S analysis combined with data from microbiome multiomic (e.g., transcriptomic, proteomic, metabolomic) analyses, data from microbiome multiomic analyses alone, and data from microbiome multiomic analyses combined with data from immunological analyses. Computational data included modeling outputs, and data from computational or statistical analyses of pre-existing data. All other data types were combined into “All other measurements”

A large fraction (approx. 62% or $452M) of these projects over this 5-year period employed 16S rRNA gene sequence analysis alone (or, in a few cases, whole genome shotgun sequence analysis) or in combination with immunological measurements or multi-omic measurements (Fig. 7). An additional 13% of the total support was for projects which collected multi-omic data alone or in combination with immunological measurements. A small proportion (6%) was for projects which used a computational approach and so used available data to conduct in silico analyses. The remaining support (19%) was for a variety of analyses of other microbial or microbial community properties.

There was a notable expansion in multi-omic data collected in these projects over this 5-year period, with the amount invested in FY2012 expanding by sevenfold by FY2016 (Fig. 7). This expansion in the variety and complexity of measurements collected in microbiome projects suggested growth in the multi-omic technologies and computational tools available to the larger research community. It also demonstrated an expansion in research focus over a relatively short period from analysis of microbial community composition to inclusion of functional properties of the microbiome.

### NIH-supported technology development for microbiome research

Though the HMP program was specifically designed to produce resources for microbiome research, the NHMPAG portfolio analysis was also assessed to estimate how many of the projects included a specific aim to develop new technologies or resources such as experimental methodologies for the study of the microbiome, new computational/statistical approaches for the analysis of microbiome data, or a microbiome-based product or device. Most of the studies in this portfolio used existing methods, such as DNA sequence analysis and the computational tools to analyze microbial community composition developed in the HMP program. Approx. 75% of the support was for projects which made use of existing HMP resources (data not shown).

**Fig. 8:**
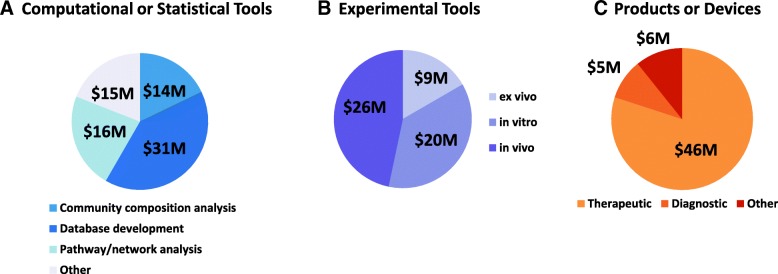
Technology development in the microbiome projects, FY2012–2016. This figure depicts the three main technology categories of computational/statistical tools, experimental tools and products/devices developed in the microbiome projects, which summed $188M over fiscal years 2012–2016. **a** Depicts computational/statistical tool development further subdivided into methods for microbial community composition analysis, microbial and microbial community metabolic pathway/network analysis and database development. Other computational analyses were combined under “Other.” **b** Depicts experimental tool development further subdivided into ex vivo, in vivo or in vitro tools. **c** Depicts product/device development further subdivided into therapeutic, diagnostic or other products/devices

However, a notable amount (approx. 26% or $188M) of the total support over this 5-year period was in the development of a specific method, tool, or product (Fig. 8). These included projects which developed new computational or statistical tools or pipelines (41%), over half of which was for the analysis of sequence data for microbial metabolic pathway prediction or for the analysis of microbial community composition (Fig. 8a). Further, a significant amount of the support for these activities (40%) went toward database development, particularly for microbial metabolic pathway analyses. A relatively small amount of this support (5%) went toward analysis of metaproteomics or metabolomics data, likely because these data types were still relatively new aspects of microbiome studies in this period of the field. In addition, the tools for analysis of these kinds of data were still relatively limited.

An additional 29% of the $188M total was in the development of in vivo (47%), in vitro (37%), or ex vivo (16%) experimental tools for microbiome research (Fig. 8b). Examples of tools categorized as in vivo included the development or refinement of alternate animal models for human microbiome research such as zebrafish or the development of probes which permit real-time measurements of such properties as dissolved oxygen levels in the gut. Examples of in vitro tools included high throughput sequence-based screening methods for the analysis of viruses or phage in blood or other tissues or development of fluorescence microscopy-based methods for the spatial analysis of biofilms or the development of organoids for use in microbiome studies. Examples of ex vivo tools included new methods for the cultivation of previously unculturable microorganisms from tissues or the development of stable isotopic methods for studying the growth and metabolism of human microbiota or high throughput screening methods for the identification of small molecules produce by the microbiota.

Finally, a similar amount (31% of $188M) was in the development of microbiome-based devices or vaccines (Fig. 8c). Most (84%) of these developments were for therapeutic products such as microbiome-based live biotherapeutic products (i.e., probiotics), prebiotic products, or dietary supplements intended for future testing to treat a specific condition or disorder or microbiome-based novel antimicrobials. Approx. 10% was for the development of diagnostic products for the analysis of microbiome-based biomarkers in disease or for the analysis of microbial metabolic products. The remainder of these studies was for the development of analytical devices or other microbiome-based products or devices. A few projects developed microbiome-derived vaccines against opportunistic pathogens such as *Clostridium difficile*.

### Selected research highlights from the portfolio analysis

This section highlights some of the resources and research advances which have resulted from this 5-year investment. Not all of the resources and advances are highlighted here but rather a sampling of studies, which illustrate some of the emerging insights into human microbiome structure and function and the roles that specific microbiome properties play in maintaining health and in disease initiation and exacerbation, are provided.

### High throughput lab approaches, access to extensive publicly available datasets and analytics

NIH’s investments during this early phase of the human microbiome field have resulted in a number of important technical advances and conceptual breakthroughs. For example, since 16S rRNA gene sequence analysis has become a routine assay, bacterial community composition is a common feature in most of these projects. As a result, there is now an extensive literature on bacterial community composition from both sexes, many different cohorts, of different ages and ethnic/racial groups, under many different disease conditions, and from a number of different body regions. Therefore, these data can now provide a view of the breadth and diversity of microbial communities across different human populations and the environmental, genetic, and cultural factors that shape the human microbiome [31, 32], something the field did not have only a few years ago. In addition, many of these datasets are available from longitudinal studies, providing a window into the dynamic features of microbial communities in different human populations, e.g., [33, 34] and across different stages of life, e.g., [35, 36].

Though still far less common than 16S rRNA gene analysis, whole genome shotgun metagenomic sequence analysis exhibited increasing use over the 5 years of this portfolio analysis period. This is partially because it was becoming more affordable and particularly because an array of computational tools for analysis of these kinds of data was becoming more broadly available, e.g., [37, 38].

Metagenomic sequence analysis also provided broader insights into these communities because not only can community composition be derived from these data but non-bacterial members of the microbiome, like phage and viruses, can be identified through metagenomics, e.g., [39, 40]. Further, these metagenomic data can also be used to evaluate the metabolic pathways encoded in the microbial genomes of the members of these communities. In particular, the analysis of microbial metabolic pathways has provided important insights into changes in community metabolism which precede or follow onset of particular diseases or in response to specific interventions, e.g., [41, 42]. Prediction of metabolic pathways from metagenomic data has also proven useful for hypothesis testing, e.g., [43] or as inputs to mathematical models, e.g., [44] .

### Development of more accurate predictive animal, ex vivo and in silico models

Animal models have been successfully used to study the functional aspects of members of the microbiota, including those with beneficial effects for the host. For instance, the anti-inflammatory properties of a probiotic strain *Lactobacillus reuteri* (*L*. *reuteri*), a result of conversion of the amino acid histidine to histamine, was recently demonstrated in a colon carcinogenesis mouse model [45]. The underlying basis for this protective effect against cancer involves a quorum sensing mechanism by which *L*. *reuteri* expresses a soluble enzyme, diacylglycerol kinase, capable of suppressing inflammation via type 2 histamine receptor activation in the luminal gut [46]. These kinds of findings highlight the potential for developing specific live biotherapeutic products from commensal microorganisms with known biological functions that may be useful in cancer and in other disease prevention strategies.

Though not yet as commonly used as mouse models, this portfolio analysis period demonstrated an exciting expansion in the development and application of alternate animal models and even non-animal models, such as bioreactors or mathematical models, developed for microbiome research. Some of the alternate animal models developed in these projects, such as the piglet, may in many respects serve as superior models for the human gut tract compared to the traditional mouse model, e.g., [47]. In other cases, the animal models were shown to be less complex and/or have shorter generation times, providing a more cost-effective means for testing hypotheses, e.g., [48]. Bioreactors are a relatively new model for biomedical research. In the microbiome field, both naturally derived and mock community-based bioreactors have become particularly useful in deciphering specific microbe-microbe interactions that result in community-level effects, e.g., [49]. The use of organoids, organ-on-a-chip or ex vivo systems extends these models to include host physiology and host tissue-microbiota interactions, e.g., [50]. In silico models are rarer still in biomedical research but over the last few years, mathematical approaches to the analysis of complex microbial communities has provided new insights into the role of key microbial members and/or specific metabolic pathways or products in these communities, e.g., [51].

### Development of new approaches for cultivation of the previously ‘uncultivable’

Though cultures of human commensal microorganisms are needed for studies of microbial physiology and metabolism, many of these microorganisms were not previously available because most earlier culture work was focused on pathogens and because traditional culture methods were used. Several research groups, particularly those that study the oral microbiome, have developed new approaches to the cultivation of the previously uncultivable members of the microbiome. For example, one group has recently succeeded in the cultivation of a human oral TM7 phylotype (TM7x), an important member of the oral microbiome with a highly reduced genome [52]. In this particular case, TM7x can only be cultivated in the direct presence of a cultivation partner (*Actinomyces odontolyticus* strain [XH001]). A similar situation exists with *Desulfobulbus oralis*, a microorganism associated with periodontitis [53]. This microorganism was first isolated via co-culture with another microorganism, *Fusobacterium nucleatum*. Unlike TM7x, which seems to require direct cell-cell contact with its cultivation partner, *D*. *oralis* was cultivated using *F*. *nucleatum* cell-free spent medium as a media supplement. These and other kinds of novel approaches to cultivation which recognize that microbes do not live in isolation in nature but share substrates and cofactors for growth, have contributed new cultured isolates and strains to the global reference database of human-associated commensal microorganisms.

### A better understanding of factors affecting microbial community structure and function

There has been great interest in the factors which affect microbial community structure and function with much attention on the role of host diet and the specific components in diet. In particular, dietary fiber appears to play a significant role in shaping gut microbiota diversity. Several studies have shown that plant-based diets which are rich in diverse fibers promote high gut microbial diversity whereas so-called “western” diets with lower total fiber content and fiber diversity promote lower gut microbial diversity. It has further been shown that these differences in specific diet composition can have long term and even intergenerational impacts on gut microbial diversity. Recently, a study demonstrated that mice fed low-fiber diets had reduced diversity in gut microbial populations and, further, some of these microbial strains were lost when offspring were continually bred on low-fiber diets over multiple generations [54]. Some of these microbial strains were only partially restored when dietary fiber was reintroduced into the chow, suggesting that fiber was a critical factor for maintaining high microbial diversity in the gut communities. As high gut microbial diversity is increasingly appreciated as beneficial for maintaining human health and low diversity is associated with diseases including obesity, inflammatory bowel disease (IBD), cancer, and many auto-immune conditions, studies of the many dietary factors which regulate gut microbial diversity will be important in future efforts to develop specific diet-based treatments for supporting and restoring gut health.

In addition, studies have shown that human microbiota structure and function are highly organized at exquisitely fine scales. This is particularly apparent in the oral and gut microbiota where highly structured microbial consortia display distinct 3-D biogeographies based upon such factors as substrate (soft vs hard tissues), host-derived nutrients, and metabolic cross-feeding between the members in the consortia. A recent study used fluorescence in situ hybridization targeting 16S rRNA gene sequences to map these structures at the micrometer scale, first in fresh supragingival plaque from human subjects [55] followed by a 15-member human gut mock community established in gnotobiotic mice [56]. In both cases, the spatial organization of these consortia is complex and reflects the cell-cell contact and metabolic interactions taking place in these consortia. These studies and others are examples of the types of models which can recapitulate the spatial relationships and ecological niches of microbial communities in the host environment. Understanding how these highly structured microbial communities function and interact with the host will be crucial to the development of targeted microbiome-based treatments.

Another example of factors which regulate microbial community function at an exquisite scale is the demonstration that the gut microbiome has a circadian rhythm which appears to be in sync with the host circadian rhythm. It has recently been demonstrated that these gut microbial diurnal variations in composition and in metabolism appear to be predominantly driven by host dietary fat content [57]. Further, this study demonstrated that the microbial metabolites produced during fermentation, specifically short-chain fatty acids, in turn appeared to regulate host circadian clock gene expression. Examples like this and others have demonstrated the extent of coordination between the microbiome and the host at all scales and that it is this kind of intimate cross-talk between the microbiome and host which plays a major role in microbial community structure and function and in host-microbiome homeostasis.

### A more comprehensive view of the various roles of the microbiome in diseases

During this 5-year period, a greater appreciation emerged of the role that the microbiome may play in host physiology and metabolism, and in the various mechanisms by which the microbiota can cause disease. Recent studies of children in Bangladesh and Malawi have provided new insights into the role of the microbiome in malnutrition and into possible microbiome-based interventions to treat malnutrition. One study demonstrated that malnutrition in Malawian children not only had a major impact on proper growth in these children but it also has a major impact on gut microbial composition and function [58]. The extent of malnutrition in these children could be tracked across a series of unique microbial signatures which were characteristic of the state of malnutrition. Further, the malnutrition-related phenotypes of these children including growth abnormalities, altered bone morphologies, and metabolic dysfunction appeared to be associated with the microbiome as these phenotypes could be transferred to recipient hosts by transplanting fecal microbiota from these malnourished children into germ-free mice, thereby recapitulating the condition. This study further demonstrated that the malnutrition phenotype could be rectified by transplanting the fecal microbiota from healthy children into malnourished children. These kinds of studies provided the proof of principle needed to demonstrate that the microbiome embodies the factors which can cause disease in humans. Further studies which resolve the specific factors in the microbiome that cause disease will lead to refined microbiome-based interventions which can be developed for treatment.

While both host genetics and environmental factors are believed to be primary drivers behind the acquisition of the microbiota, in the case of the oral microbiome and diseases such as dental caries, the factors driving the progression of dental caries are largely unknown. In a first ever analysis of the supragingival plaque microbiome of 485 dizygotic and monozygotic twin pairs of children, it was revealed that although some of the oral microorganisms were heritable, most of the potentially cariogenic species were environmentally derived [59]. Further, the heritable microbial species decreased in abundance as the children aged and the oral microbiome became dominated by environmentally derived microbial strains, including potentially cariogenic strains. This and other such studies were the first to evaluate the internal versus external sources of microorganisms in the microbiome which can cause disease.

Unlike dental caries, other microbiome-related diseases appear to be associated with commensal members of the microbiome. These disease-causing commensal microorganisms are known as pathobionts because specific conditions can trigger a normally benign or even beneficial microorganism to overgrow its habitat and become pathogenic. One example of this involves inflammatory bowel disease (IBD) and its disease subtypes, Crohn’s disease, and ulcerative colitis. In IBD, some members of the normal gut microbiota expand in numbers while other members decline [60]. Though many factors appear to contribute to the overgrowth of these microorganisms [61], it is thought that the overgrowth of these normally commensal members leads to dysregulation of mucosal immunity and a disruption of gut barrier function [62]; repeated cycles of these expansions along with inflammation lead to flares. These kinds of studies lay the groundwork for the development of subsequent specific treatment strategies for classes of disease such as caries which would specifically target environmentally derived microorganisms versus classes of disease like IBD which would prevent the commensal members from becoming pathobiotic.

Finally, some microbiome-associated diseases appear to be the result of translocation of a microorganism from its normal habitat into other tissues, thereby setting up an inflammatory cascade that can trigger disease. An example of this can be found with lupus, an autoimmune disease. One study demonstrated that when a commensal gut microorganism, *Enterococcus gallinarum*, translocated from the gut to the liver and other tissues, it appears to trigger a suite of autoimmune responses which led to lupus in genetically susceptible subjects [63]. In this study, *E*. *gallinarum* was found in liver biopsies of lupus patients but not in healthy controls. Further, in the same study, these observations were verified in a mouse model where those mice with lupus-like conditions had *E*. *gallinarum* in the liver and lymphoid tissues and pathogenic T helper cells were found in these tissues. Future treatment strategies which address diseases caused by translocation of microorganisms from normal tissues would focus on the prevention of these translocation events such as improving gut barrier function rather than on the eradication of these microorganisms.

### Development of microbiome-based biomarkers for disease risk and detection

One area which has seen some advances is the development of microbiome-based biomarkers for predicting disease risk. These emerging biomarkers generally range from the presence or absence of specific bacteria to an altered composition of the microbiome compared to the general population to the presence or absence of specific microbial metabolites or a suite of metabolites. For example, an altered gut microbiome appears to be indicative of children at risk for type 1 diabetes [64] or asthma [65].

With respect to specific strains of bacteria as biomarkers, the absence of *Christensenella minuta*, one of the most heritable microorganisms in the gut microbiome, appears to be correlated with high body mass index (BMI) [66]. In colorectal cancer, the presence of a number of specific bacterial taxa (*Parvimonas micra*, *Streptococcus anginosus*, and some uncultured members of the Proteobacteria) in the gut microbiome appears to be a biomarker for this disease [67]. The presence of specific strains of *Gardnerella vaginalis* and the absence of *Lactobacillus crispatus* were strong indicators for pregnant women at risk for preterm birth, especially for African-American women [68].

One of the earlier studies which demonstrated that microbial metabolites could serve as useful biomarkers of disease was in cardiovascular disease. Tri-methylamine *N*-oxide and phosphatidylcholine, two microbial metabolites produced during the digestion of meat, appear to be strong biomarkers for patients at risk of atherosclerosis [69, 70]. In a very interesting example, a suite of microbial metabolites in the gut microbiome of children with autism spectrum disorder (ASD) appeared to correlate with different forms of ASD and could even be used to categorize the type of ASD the child exhibited [71]. In the same study, there was also evidence of different concentrations of neurotransmitters in the stool of ASD children compared to neurotypical children, suggesting an independent line of investigation of microbially mediated neurotransmitters for studying this childhood condition.

### Development of innovative microbiome-based prevention and intervention strategies

Though this field is still in its early days, development of a number of microbiome-based disease preventions and interventions are already underway, some of which are being evaluated by the FDA. Fecal microbiota transplantation (FMT) shows efficacy for recurrent *C*. *difficile* gut infections but has not yet shown reproducible efficacy for other conditions. Further, FMT is acknowledged as a “black box” approach for treating disease so more precise strategies have been under development. These strategies currently fall into four general categories: (a) host microbiota-derived live biotherapeutic products for reducing inflammation, restoring gut barrier function, or, in some cases, improving colonization resistance against pathogens; (b) treatments which target-specific pathogens using microbiome-sourced antibiotics that can target antibiotic-resistant pathogens as well as the use of narrow host-range phage; (c) prebiotics which are microbial growth substrates that can stimulate the metabolism and growth of specific commensal microorganisms; and (d) specialized metabolites (such as microbial signaling molecules) or bacterial cellular components which can regulate or limit specific microorganisms or act directly on host pathways. A few examples of these promising strategies developed during the portfolio analysis period will be noted here.

In the cancer field, an exciting area of research that has emerged is the role played by host-microbial interactions, particularly microbial metabolite signals, in regulating host inflammatory responses and tumor immunity [72]. This work has important implications for cancer prevention, etiology, and particularly treatment, as indicated by recent work which demonstrates that when gut bacterial communities are compromised, chemo and immunotherapies regimens may lose efficacy, e.g., [73–75]. Understanding the critical role that commensal microbiota play in modulating host immune responses may help to explain the clinically observed variation in patient response to immunotherapies and aid efforts to optimize personalized medicine regimens by incorporating information from a patient’s microbiome status as a part of the treatment plan.

In the spectrum of conditions of the related diseases of metabolic syndrome-insulin resistance-obesity, many approaches have been tested to alleviate these conditions. Earlier work showed that changes in the gut microbiome were associated with these conditions but the mechanisms of interaction were not known. More recent work has demonstrated that short-chain fatty acids, generally considered important microbial metabolites and host signaling molecules, can when over-produced, lead to a complex cascade of undesirable responses in the host and ultimately to obesity [76]. This study demonstrated that mice fed a high-fat diet overproduced one of the key short-chain fatty acids (acetate) which in turn led to an overstimulation of the parasympathetic nervous system that promoted increased glucose-stimulated insulin secretion, and ghrelin secretion which ultimately led to weight gain. This study demonstrated a mechanistic link between microbially mediated metabolites produced in the gut and overstimulation of the signaling pathway in the brain which controls appetite and weight gain. The results of this study pointed to new modes of action for insulin secretion and thereby offered potentially new interventions based on regulation of diet and/or of the acetate-producing microorganisms in the gut.

Finally, many studies have shown that not all patients with the same disease or condition respond similarly to treatment and particularly so in the case of drug treatment. In many cases, microbial metabolism of or the effect of a drug on the microbiome may be related to patient response to treatment. Not only does the gut microbiome metabolize many kinds of drugs but, in many cases, microbial metabolism is relied upon for drug activation, e.g. [77]. Further, the drugs themselves can directly affect microbial metabolism and alter community composition [78]. These microbiome-drug interactions are now being considered and even leveraged, such as is being done with cancer immunotherapy, in the development of future intervention strategies.

It is clear there is no “one size fits all” strategy for microbiome-based interventions for treating disease. Each intervention strategy will need to take into account the class of disease and the specific role of the microbiome in it, the individual subject variation in microbiome composition and in microbial community metabolism, issues of colonization resistance and in some cases, the role of host genetics in these interventions.

### Gaps and opportunities which emerged from this portfolio analysis

This section completes the portfolio analysis by highlighting some of the technical needs and knowledge gaps which remain for this field to be able to advance over the next decade and so that the outcomes from human microbiome research can ultimately be incorporated into efforts to support health and treat disease.

### New approaches to study microbe-microbe interactions

There is still much to understand about how microbe-microbe, inter-kingdom microbial interactions, and microbial community level interactions occur, and how these interactions may play a role in human health and disease. Some of these knowledge gaps would benefit from studies of cultured microorganisms of human-associated microbes but these are still not broadly available. For example, many microbial members identified in the HMP healthy cohort metagenomic reference database do not yet have known cultured representative strains or isolates [79], and this presents a significant technological gap for microbial physiology studies. This issue is being addressed to some degree in the oral microbiome field through new methods for laboratory cultivation of oral taxa. However, a broader range of microbiological and engineering approaches are needed to isolate and cultivate representative members of the human microbiome.

In addition, there is limited understanding of the other members of the human microbiome outside of the bacteria, such as bacteriophage, viruses, and fungi, all of which are likely to play important roles in the human host. For example, bacteriophage may play major roles in the microbial community composition and function due to their ability to regulate the genes in the microbial hosts they infect and their ability to mediate horizontal gene transfer between host bacteria. Whole genome shotgun metagenomics, coupled with sample fractionation methods, are some approaches which can provide a catalog of the phage and viruses from a body region. However, new approaches are needed which can yield culturable isolates of human-associated phage and viruses for subsequent study.

Finally, little is known about the nutrient requirements or other factors (such as specific metabolites) which may limit or regulate the microbial members in these communities, which in turn would play a role in our interpretations of the microbiome in disease initiation or exacerbation. For example, studies have shown that certain secondary bile acids can regulate the sporulation and growth of *C*. *difficile*, a member of the GI microbiome that causes severe GI disease. This mechanistic insight into *C*. *difficile* sporulation and growth can serve as a model for studying the factors which regulate the metabolism and growth of not only opportunistic pathogens but also commensal members of the microbiome. Approaches which can take advantage of functional genomics and metabolomics would yield important new insights into these factors. Novel approaches which use radiotracers, stable isotopes, or metabolic inhibitors may also lead to new understanding of specific nutrient requirements, limiting factors, or other factors related to the food web of these microbial communities. Such studies could also present a new paradigm for the discovery and rational design of microbiome-based therapeutics. Further, surprisingly little attention was paid to the role of antimicrobial resistance in the regulation of these microbial communities, an ussue which needs to be included in future work on microbiome-based therapeutics.

### New approaches to study microbial associations with specific host tissues

Though bulk stool is commonly used to sample the GI tract, it is known that the microbial composition and cell abundance varies greatly along its length and width. In fact, it is still an open question whether stool can serve as a reasonable proxy for the GI tract. In some studies, the GI tracts of mice and other vertebrate animal models have been dissected in order to evaluate the spatial biogeography of microorganisms associated with different immunologically important tissues as well as to examine the distribution of luminal versus lymphoid tissue-associated microbial populations. In addition, a few human cohort studies have sought to compare the microbial composition in stool with brush or biopsy collected mucosal-associated samples. More such studies are needed to establish the range of conditions under which stool can serve as a proxy for the GI tract microbiome. These kinds of body site-specific analyses are needed not only for the GI tract but also for other body regions, such as the oral and urogenital (i.e., vaginal, penile) body regions, to inform epidemiological studies of the most appropriate sample type for use in microbiome analyses.

Further, new approaches are needed for in situ sampling of microbial communities associated with specific host tissues in difficult-to-sample body regions and for measuring their functional activities in order to evaluate the role of different microbial communities in the different habitats of a body region. This holds true not only for the GI tract but also for other body regions where multiple microbial habitats occur in a body region such as the oral and urogenital body regions.

Finally, though bulk analysis of microbial communities is a routine method in current microbiome studies, it is not yet clear if all the microorganisms in a bulk sample can be treated the same or, more importantly, if they can be treated as if they play the same role in the host. For example, some studies of the GI tract microbiome have suggested that only certain members interact directly with the host while other members may play more of a role in maintaining the microbial habitat and not necessarily interact directly with the host. New approaches which can differentiate host tissue-associated microorganisms from the other members of the microbial community may provide new insights into the presence of different ecological niches in specific body regions and the roles of specific members in host-microbe interactions. These kinds of studies would especially benefit from collaborations with immunologists and gastroenterologists with expertise in mucosal tissue biology.

### New approaches to study the role of host genetics in the microbiome

More attention should be paid to the role of host genetics in microbiome assembly in host-microbiota interactions and in disease. There remains debate in the literature with some studies suggesting that host genetics plays a major role in the assembly of the infant gut microbiome with the environment playing a greater role in adult subjects so that the microbiome is considered a partially heritable trait. On the other hand, some studies have demonstrated interactions between host genetics, some members of the microbiome and factors such as diet, innate immunity, vitamin D receptors, as well as some autoimmune diseases. New approaches are needed to establish the conditions under which host genetics plays a role in microbiome structure and function as these kinds of data would be useful, for example, for stratifying subjects in cohort studies of disease risk or for testing treatments or interventions.

## Additional file

Additional file 1: Supplemental Information. Methods and Writing Team Institute Affiliations and Contact Information. (DOCX 31 kb)

## Footnotes

1 The US federal government fiscal year is October 1 to September 30.
