# Supplementary material for: A review of 10 years of human microbiome research activities at the US National Institutes of Health, Fiscal Years 2007-2016
Source: Microbiome. 2019 Feb 26;7:31. doi: 10.1186/s40168-019-0620-y (PMC6391833; doi:10.1186/s40168-019-0620-y)
Supplement: Supplementary file 1 — Supplemental Information. Methods and Writing Team Institute Affiliations and Contact Information. (DOCX 31 kb) [file 40168_2019_620_MOESM1_ESM.docx]

# **Supplemental Information**

## **Methods**

The TMWG committee developed a customized spreadsheet to collect and analyze the microbiome research activities supported by the NIH extramural program. The NIH’s Query, View, Reporting (QVR;<https://archives.nih.gov/asites/era/11-25-2015/nih_and_grantor_agencies/other/query_view_and_report.cfm>) database was used for developing a parent list of grants over fiscal years 2012-2016. QVR was used instead of NIH RePORTER (<https://projectreporter.nih.gov/reporter.cfm>) for the initial query to capture more properties about each grant than is typically available in the RePORTER database. Though this portfolio analysis cannot be specifically recreated, all of the information in this portfolio analysis are available on the NIH RePORTER database.

For this analysis, a microbiome is ‘a multi-species population or community of microbes in a specific host or environment’ and includes bacteria, archaea, fungi, eukaryotic viruses and bacteriophage while microbiome research is the study of microbial community composition, structure and function, microbe-microbe interactions or interactions with their hosts in research related to human health or disease. A project is defined as any annual research activity which is described in the specific aims of an application and has the same base NIH grant number. A grant is a research activity which is supported over one funding cycle; for example, an investigator-initiated grant typically has a five-year funding cycle. An award is the funding provided each year to a grant or supplement to a grant.

The global search terms ‘microbiota’ and ‘microbiome’ were used to identify potentially relevant projects in QVR. This parent query of over 3200 entries was then manually curated by reviewing the abstracts and specific aims of each award to identify if the activities specifically described microbiome research, based on the committee’s definition of microbiome research. This filtered list of approximately 2700 entries, each now counted as a project, served as the master spreadsheet for further curation.

The pool of investigators in this analysis was determined by summing the unique investigators in single PI awards and in multi-PI awards for research, center and training awards for each year of the portfolio analysis period. HMP-funded investigators were counted separately from all other NIH-funded investigators.

The master spreadsheet was distributed to the TMWG member of each NIH Institute, Center or Office (IC) which supports microbiome research to classify the projects into four general categories of single principal investigator grants, center grants, training grants or meeting grants. The grants were also categorized into two classes of NIH grants: type 1s grants (e.g. new grants) or type 2s (e.g. competing renewal grants). Though information on specific investigators, institutions, funding opportunity announcements, NIH program directors and ICs was available in the NIH database, none of this information was included in the final analysis.

The projects were further curated to include the total funding for microbiome research, apportioned based on the specific aims, whether the research involved human cohort studies, animal models, or some combination thereof, whether the studies that involved animal models used vertebrate or invertebrate animals, nonhuman primates or some combination thereof, and of those studies which involved human subjects or animals, which region(s) of the body was studied. There were fourteen body regions and tissues to choose from including blood, cardiovascular, central nervous system, ear, eye, gastrointestinal tract, lung, nares, oral, skin, urogenital, or other region or whole body or multiple body regions.

The projects were classified according to six high-level microbiome themes, including larger community interactions, microbe-microbe interactions, biofilms, microbial products and specific microbe. Though culture studies were generally not included in this analysis, an example of a microbiome project which focused on a specific microbe might be a project that examined short chain fatty acid production by a specific microbe and how this specific microbial metabolism affected the larger microbial community. Further classification of these projects noted if the primary microbial component under study were archaea, bacteria, fungi, viruses (both eukaryotic viruses and bacteriophage) or microbial products.

Information on the primary research focus of the project was collected. Each project was classified by whether the work was primarily focused on fundamental aspects of the microbiome without regard to a specific disease or if disease-focused, what class of disease was under study. The projects which did not address disease were classified into one of six areas of emphasis (colonization, evolution, immune system, microbial signaling, physiology/metabolism or ethical, legal, social implications - ELSI). For the disease-focused projects, the chapters and blocks in the World Health Organization’s International Catalog of Diseases, version 10 ([www.who.int/classifications/icd/en](http://www.who.int/classifications/icd/en)) was used to classify diseases.

The committee analyzed information on the kinds of microbiome data proposed to be collected in each project. Information on whether primarily 16S rRNA and/or whole genome shotgun sequence data was collected for the project was noted. Other choices including the collection of sequence data with immunological measurements and/or multi-omic measurements like transcriptomic, proteomic or metabolomic data. The collection of primarily immunological, metabolomic or host response data, for example in studies where specific microbiome-based interventions were being tested, was also noted.

Finally, an effort was made to determine if a project included technology development, such as development of a product, or an experimental, computational or statistical tool. A product was classified as a therapeutic, diagnostic or a vaccine or prophylactic product. An experimental tool was classified as *in vivo*, *ex vivo* or *in vitro* tool. A statistical tool was further classified as a tool for analysis of community composition, proteomic or metabolomics data. A computational tool was further classified as a tool for DNA/RNA, community composition, pathway/network, proteomic or metabolomic data analysis. In addition, statistical or computational tools could also be for database development. It was assumed that a project that did not mention technology development in the specific aims made use of published methods or tools.

Once the initial curation was completed by the IC program staff, the master list of projects was reviewed for completeness and clarity. This involved several ‘blinded’ analyzes of subsets of the 2700 entries to verify that the same results were obtained and if not, the committee discussed any disparities and possible solutions to them. In addition, several rounds to correct any blank cells or erroneous entries were conducted. No information on the name of a specific principal investigator(s), institution, state, funding opportunity announcement, NIH program director or IC was included in this analysis. Therefore, none of these potentially identifiable properties are reported in the graphs or tables for this analysis or discussed in the paper. All data were anonymized with respect to grantee, institution, funding opportunity announcements or NIH Institute or Center.

**NHMPAG co-author contributions**
